# The Recovery of Thyroid Function in Low-Risk Papillary Thyroid Cancer After Lobectomy: A 3-Year Follow-Up Study

**DOI:** 10.3389/fendo.2020.619841

**Published:** 2021-02-09

**Authors:** Yi Dou, Yingji Chen, Daixing Hu, Xinliang Su

**Affiliations:** Department of Endocrine and Breast Surgery, the First Affiliated Hospital of Chongqing Medical University, Chongqing, China

**Keywords:** papillary thyroid cancer, hypothyroidism, thyroidectomy, thyrotropin, lobectomy

## Abstract

**Purpose:**

Management strategies after lobectomy for low-risk papillary thyroid carcinoma (PTC) are controversial. This study aimed to identify the proportion of patients among low-risk PTC patients who do not require hormone replacement therapy and to evaluate the risk factors for postoperative hypothyroidism after lobectomy.

**Patients and Methods:**

The records of 190 PTC patients who underwent thyroid lobectomy from January 2017 to December 2018 were retrospectively reviewed. Clinicopathological characteristics and follow-up data were collected. Univariate and multivariate analyses were performed to identify the risk factors associated with postoperative hypothyroidism and the recovery of thyroid function.

**Results:**

In summary, 74.21% of patients (141/190) had normal thyroid function without levothyroxine supplementation, while 40.53% (77/190) developed temporary or permanent hypothyroidism. Multivariate analysis indicated that higher preoperative thyroid-stimulating hormone (TSH) levels (>2.62 mIU/L), Hashimoto’s thyroiditis (HT), and right lobectomy were associated with hypothyroidism (all P<0.05). The Area Under Curve (AUC) by logistic analysis was 0.829. Twenty-eight (28/77, 36.4%) patients recovered to the euthyroid state in the first year after surgery, and this recovery was significantly associated with preoperative TSH level. Forty-nine (49/77, 63.6%) patients developed persistent hypothyroidism. The thyroid function of most patients (11/28, 39.3%) recovered in the third month after surgery.

**Conclusion:**

Patients with a lower level of preoperative TSH, with left lobectomy and without Hashimoto’s thyroiditis had a higher chance of normal thyroid function within the first year after lobectomy. The recovery of thyroid function was associated with the level of preoperative TSH.

## Introduction

Thyroid cancer is the sixth most common malignancy in women (1), and its incidence has rapidly increased in recent years. Lobectomy is recommended as a standard initial treatment for low-risk papillary thyroid carcinoma (PTC) in both the National Comprehensive Cancer Network (NCCN) (2) and European Society for Medical Oncology (ESMO) guidelines (3). The American Thyroid Association (ATA) guidelines (4) also provide a strong recommendation for lobectomy in patients with thyroid cancer <1 cm. Even experienced surgeons cannot avoid complications caused by expansion of the operative extent in total thyroidectomy. Furthermore, lobectomy patients can benefit from fewer complications (5), such as hypocalcaemia or injury of the recurrent laryngeal nerve (6). Moreover, the overall survival (7) and disease-free survival (DFS) (8) rates are similar between patients who undergo lobectomy and total thyroidectomy, as reported by previous studies. However, the management of postoperative thyroid function remains controversial among patients. Most studies have shown that 9%–64.2% of patients develop hypothyroidism after lobectomy (9–11). This controversy comes from differences in the definition of hypothyroidism, patient follow-up time, and the duration of thyroid hormone replacement therapy (12).

Due to the widespread use of thyroid hormone therapy in the clinic, patients begin to take thyroid hormone in the early postoperative period. However, monitoring thyroid function in patients interferes with thyroid hormone therapy. Moreover, thyroid hormone therapy has many side effects in patients with long-term hyperthyroidism/subclinical hyperthyroidism as a result of long-term overdose and failure to provide regular monitoring. The incidence of kidney, pancreatic, ovarian, and breast cancers was found to increase with long-term iatrogenic hyperthyroidism according to a large-sample epidemiological study in Norway (13). A cohort study (14) also described the association between thyroid-stimulating hormone (TSH) levels and risk of cardiovascular disease.

The purpose of this study was to determine the proportion of patients who do not need hormone replacement therapy and to determine its predictors of this outcome by following patient thyroid function. The relative factors and average time of recovery to the euthyroid state were analyzed to determine the surgical strategy and management of postoperative thyroid function in patients with low-risk thyroid carcinoma.

## Materials and Methods

### Patients and Study Design

A total of 253 patients who underwent unilateral lobectomy due to PTC at the Department of Endocrine and Breast Surgery of the First Affiliated Hospital of Chongqing Medical University from January 2017 to December 2018 were enrolled in this retrospective analysis. Patients with hyperfunctioning thyroid adenoma (n=5), other types of carcinoma (n=5), lymphatic positivity (n=11), hypothyroidism before operation (n=14) or a lack of follow-up data (n=28) were excluded. Based on these criteria, 190 patients with PTC were included. Consent was obtained from each patient after full explanation of the purpose and nature of all procedures. The study was approved by the Medical Ethics Committee of The First Affiliated Hospital of Chongqing Medical University (2020-219).

### Clinicopathological Variables

**Figure 1:**
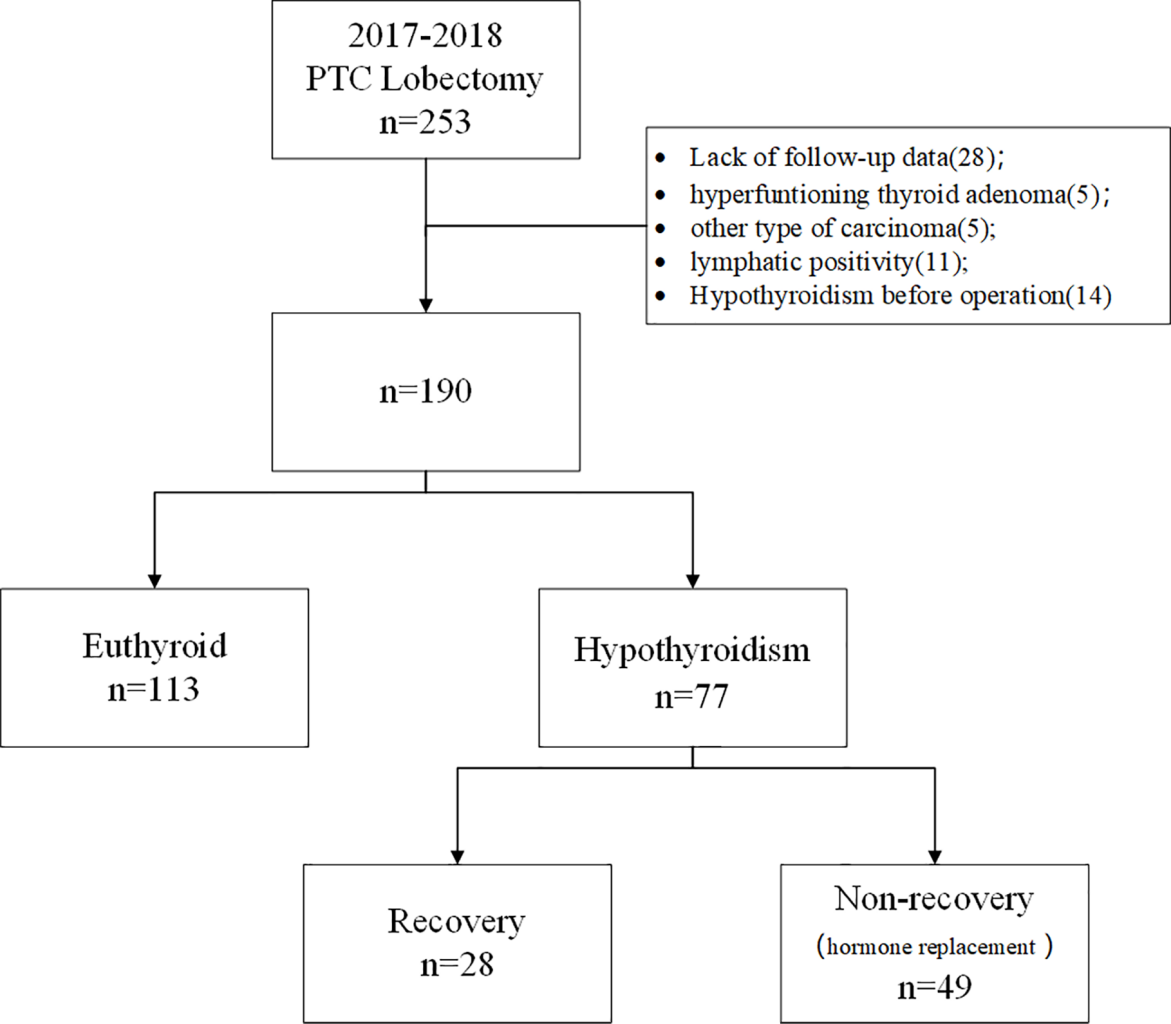
Study flowchart.

Age, sex, tumor size (maximal diameter), tumor location (upper/middle/lower pole), body mass index (BMI), resection of the left/right lobe, Hashimoto’s thyroiditis (HT), preoperative TSH, and postoperative thyroid function at every follow-up visit were recorded as the patient clinicopathological variables. The diagnoses of PTC and HT were confirmed by pathological examination. Patients with normal preoperative TSH levels were divided into two groups (euthyroid group vs. hypothyroidism group) based on the levels of postoperative TSH, serum free thyroxine (fT4), and total serum thyroxine (TT4). Within the hypothyroidism group, patients were enrolled in the recovery group if their thyroid function returned to a normal level without hormone intake. Otherwise, they were enrolled in the group of patients who did not recover to normal thyroid function (non-recovery), which included patients who received thyroid replacement therapy and could not stop hormone dependence (n=49) (**Figure 1**).

### Surgical and Follow-Up Procedures

All patients were diagnosed *via* fine-needle aspiration biopsy (FNAB) before surgery. Lymph node status was estimated by two experienced ultrasound doctors. Thyroid function and thyroid autoantibodies were tested before operation. Patient treatment and risk factor assessments were made in accordance with both the ATA and Chinese guidelines, and the scope of surgery was at least lobectomy on the affected side and central lymph node dissection of the affected side. Low-collar incision minimally invasive lobectomy with unilateral central compartment lymph node dissection (LND) was performed in those patients. Intraoperative frozen biopsy was routinely performed in each patient to determine the extent of surgery. The contralateral thyroid lobe was retained only if frozen biopsy confirmed no lymph node involvement. In this way, the risk of recurrence in the contralateral thyroid lobe was minimized. Therefore, we did not immediately administer hormone therapy to these patients. Patients were followed in the first month, every three months during the first postoperative year, and then every six months during the second and third years. Thyroid function and related symptoms were carefully evaluated. Neck ultrasonography was performed for suspected recurrence and metastasis at each follow-up visit. During our follow-up period (20-36 months), no patient showed definite cervical lymph node or residual thyroid lobe recurrence. Patients with TSH levels >10 mIU/L (significantly higher risk of cardiovascular mortality and morbidity) or TSH levels >5.9 mIU/L with serious intolerable hypothyroid signs, such as severe asthenia, myxoedema or heart failure, were given L-thyroxine (15). The dosage of thyroid used for replacement was 50–150 µg/day, and the dosage was adjusted according to the patient’s thyroid function laboratory tests. We would reduce the levothyroxine dose by 25 µg at each follow-up time. When patients fell into a state of clinical hypothyroidism, or subclinical hypothyroidism with hypothyroid symptoms, the levothyroxine dose would be increased appropriately. When patients showed subclinical hypothyroidism without hypothyroid symptoms, they were transiently observed without immediate hormone therapy until the other follow-up examination. The dose would be increased if their TSH levels rose continually. Otherwise, thyroid function was continuously monitored without hormone replacement therapy.

### Laboratory Measurements

All blood samples were tested by two experienced laboratory doctors. Thyroid function laboratory tests included tests of free triiodothyronine (fT3), total triiodothyronine (TT3), free thyroxine (fT4), total thyroxine (TT4), TSH, thyroglobulin (TG), and anti-thyroglobulin antibodies (TGAb) levels. All parameters were tested at each follow-up visit. The normal serum reference ranges for TSH, fT4, TT4, Tg, TgAb, and thyroperoxidase autoantibody (TPO) from our Clinical Laboratory Department were 0.56–5.91 mIU/L, 0.59–1.25 ng/dl, 5.44–11.85 µg/dl, 5–50 μg/L, 0–4 mIU/L, and 0–9 mIU/L, respectively. Hypothyroidism included subclinical hypothyroidism (15), which was defined as abnormal TSH values and FT4 and fT4 values within the reference range (TSH>5.91 mIU/L with normal fT4 and TT4 levels in our hospital), and clinical hypothyroidism, which was defined as abnormal TSH values with decreased fT4 or TT4 (TSH>5.91 mIU/L with fT4<0.59 ng/dl or TT4 <5.44 µg/dl in our hospital). No patient was pregnant during the perioperative period or the whole follow-up period.

### Statistical Analyses

Univariate and multivariate data were analyzed using SPSS version 23.0 (SPSS Inc., Chicago, IL, United States). Student’s t-test was used to examine continuous variables, which were described as the means with standard deviations. The x^2^ test was performed to analyze the categorical variables. Receiver operating characteristic (ROC) curve analysis was performed to analyze the cut-off value for the predictive ability of preoperative TSH for postoperative hypothyroidism. Logistic regression analysis was used to analyze multivariate variations between the euthyroid group and hypothyroidism group and between the recovery group and the non-recovery group. Survival curves describing thyroid function recovery during follow-up were analyzed by Kaplan-Meier analysis. A two-tailed P-value below 0.05 was used to indicate statistical significance. Both the thyroid function curves for the different subgroups and the histograms were prepared with GraphPad Prism 8.0 (GraphPad Software Inc., San Diego, CA, USA).

## Results

### Clinicopathological Patient Characteristics

Among the total of 190 patients, 113 (59.47%) patients remained in the euthyroid state during the whole follow-up period after operation, while 77 (40.53%) developed temporary or permanent hypothyroidism. The sex characteristics of the 190 patients (140 (73.7%) females and 50 (26.3%) males) are presented in **Table 1**. The average age at diagnosis was 40.4 ± 10.4 years, the average tumor size was 0.8 ± 0.6 mm, and the average BMI was 22.9 ± 3.2. The preoperative TSH, TPO, TgAb, and Tg levels were 2.4 (0.7–5.7) mIU/L, 0.8 (0.0–971.0) mIU/L, 0.1 (0.0–877.0) mIU/L, and 7.0 (0.2–464.0) μg/L, respectively. By univariate analysis, there was no significant difference in mean age, tumor size, sex, tumor location, BMI, TPO, or Tg between the two groups. More right lobectomy patients were hypothyroid (70.1% vs. 49.6%), and this difference was significant (P=0.005). High levels of preoperative TSH, high levels of TgAb, and HT were significant factors for postoperative hypothyroidism (P< 0.05 for all comparisons).

**Table 1:** Univariate analysis of factors associated with euthyroid group and hypothyroid group in 190 patients with euthyroid before operation.

	Totaln=190	Euthyroid group	Hypothyroid group	P-value
		113	77	
Ages, years	40.4 ± 10.4	40.2 ± 9.9	40.9 ± 11.2	0.777
<55	175 (92.1%)	105 (92.9%)	70 (90.9%)	0.614
≥55	15 (7.9%)	8 (7.1%)	7 (9.1%)	
Size of tumor, cm	0.8 ± 0.6	0.8 ± 0.7	0.8 ± 0.5	0.992
≤1	158 (83.2%)	96 (85.0%)	62 (80.5%)	0.422
>1	32 (16.8%)	17 (15.0%)	15 (19.5%)	
Sex				0.077
Female	140 (73.7%)	78 (69.0%)	62 (80.5%)	
Male	50 (26.3%)	35 (31.0%)	15 (19.5%)	
Resection of Left/Right				0.005
Left	80 (42.1%)	57 (50.4%)	23 (29.9%)	
Right	110 (57.9%)	56 (49.6%)	54 (70.1%)	
Location				0.835
upper	61 (32.1%)	38 (33.6%)	23 (29.9%)	
middle	82 (43.2%)	47 (41.6%)	35 (45.5%)	
lower	47 (24.7%)	28 (24.8%)	19 (24.7%)	
BMI	22.9 ± 3.2	22.9 ± 3.2	22.7 ± 2.9	0.870
TSH, mIU/L	2.4 (0.7–5.7)	1.9 (0.7–5.0)	3.4 (0.8–5.7)	<0.001
TPO, mIU/L	0.8 (0.0–971.0)	0.8 (0.0–971.0)	0.8 (0.1–880.0)	0.851
TgAb, mIU/L	0.1 (0.0–877.0)	0.1 (0.0–243.0)	0.2 (0.0–877.0)	0.045
Tg, μg/L	7.0 (0.2–464.0)	6.4 (0.2–464.0)	8.2 (0.2–88.0)	0.107
HT				
NO	178 (93.7%)	111 (98.2%)	67 (87.0%)	0.002
YES	12 (6.3%)	2 (1.8%)	10 (13.0%)	

BMI, Body Mass Index; TSH, thyroid-stimulating hormone; TPO, thyroperoxidase autoantibody; TgAb, anti-thyroglobulin; Tg, thyroglobulin; HT, Hashimoto’s thyroiditis.

**Figure 2:**
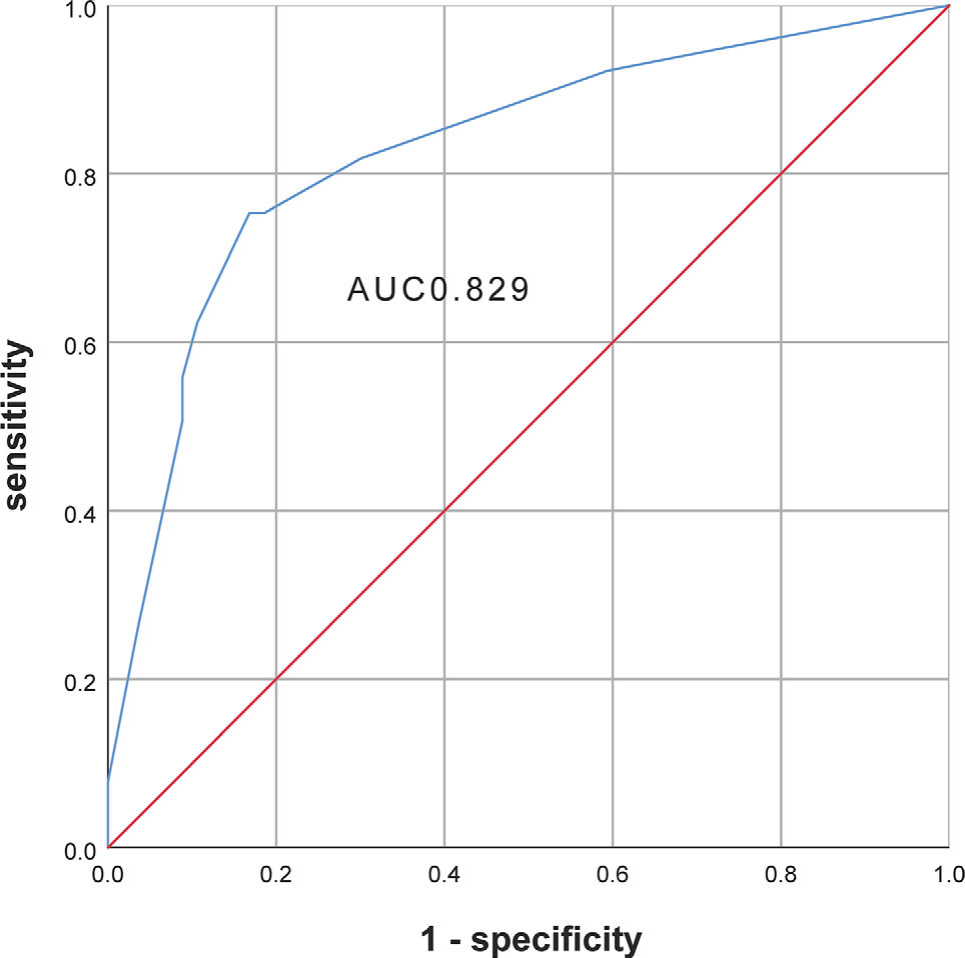
The receiver operating characteristic (ROC) curve of multivariate analysis.

The optimum cut-off value of preoperative TSH, as analyzed by the ROC curve, was 2.62 mIU/L, with a sensitivity of 0.832 and specificity of 0.701. Multivariate analysis (**Table 2**) indicated that a higher preoperative TSH level (>2.62 mIU/L) was associated with hypothyroidism (OR = 12.567, 95% CI = 6.009 to 26.285; P<0.001). HT was the second independent risk factor, with an OR of 9.293, followed by right lobectomy. TgAb was not associated with the development of postoperative hypothyroidism. **Figure 2** shows the ROC curve from the logistic analysis, indicating an AUC of 0.829 (95% CI 0.735 to 0.862).

**Table 2:** Multivariate logistic regression for predictive factors in relation to postoperative hypothyroidism in 190 patients with euthyroid before operation.

	β (SE)	P-value	OR	95% CI	
				Lower	Upper
TSH>2.62 mIU/L	2.531	0.001	12.567	6.009	26.285
preTgAb(+)	0.285	0.473	1.330	0.610	2.897
HT(+)	2.229	0.014	9.293	1.561	55.326
Right-lobectomy	0.877	0.021	2.404	1.139	5.077

TSH, thyroid-stimulating hormone; TgAb, anti-thyroglobulin; CI, confidence interval; OR, odds ratio.

### Development of Hypothyroidism

In the hypothyroidism group, 28 (36.36%) patients recovered to the euthyroid state. Forty-nine (63.64%) patients, who ultimately took hormone replacement therapy, developed hypothyroidism and did not recover during the follow-up period. **Table 3** shows that there was no significant difference in age, tumor size, sex, tumor location, or preoperative BMI, TPO, TgAb, Tg, or HT. Only a higher preoperative TSH level significantly associated with non-recovery hypothyroidism (P=0.014), and this significant difference was also found by multivariate analysis.

**Table 3:** Univariate analysis between recovery group and non-recovery group.

	recovery group	non-recovery group	P-value
	28	49	
Ages, years	40.1 ± 11.9	41.3 ± 10.9	0.302
<55	25 (89.3%)	45 (91.8%)	0.708
≥55	3 (10.7%)	4 (8.2%)	
Size, cm	0.9 ± 0.5	0.8 ± 0.5	0.582
Sex			0.128
Female	20 (71.4%)	42 (85.7%)	
Male	8 (28.6%)	7 (14.3%)	
Resection of Left/Right			0.082
Left	5 (17.9%)	18 (36.7%)	
Right	23 (82.1%)	31 (63.3%)	
Location			0.511
upper	10 (35.7%)	13 (26.5%)	
middle	13 (46.4%)	22 (44.9%)	
lower	5 (17.9%)	14 (28.6%)	
BMI	23.0 ± 2.6	22.5 ± 3.1	0.275
TSH, mIU/L	2.5 (1.4–5.1)	3.5 (0.8–5.7)	0.014
TPO, mIU/L	1.4 (0.1–552.0)	0.8 (0.1–880.0)	0.212
TgAb, mIU/L	0.1 (0.1–380.0)	0.2 (0.0–877.0)	0.199
Tg, μg/L	9.5 (0.6–88.0)	8.0 (0.2–36.7)	0.206
HT			
YES	24 (85.7%)	43 (87.8%)	0.653
NO	4 (14.3%)	6 (12.2%)	

BMI, Body Mass Index; TSH, thyroid-stimulating hormone; TPO, thyroperoxidase autoantibody; TgAb, anti-thyroglobulin; Tg, thyroglobulin; HT, Hashimoto’s thyroiditis.

**Figure 3:**
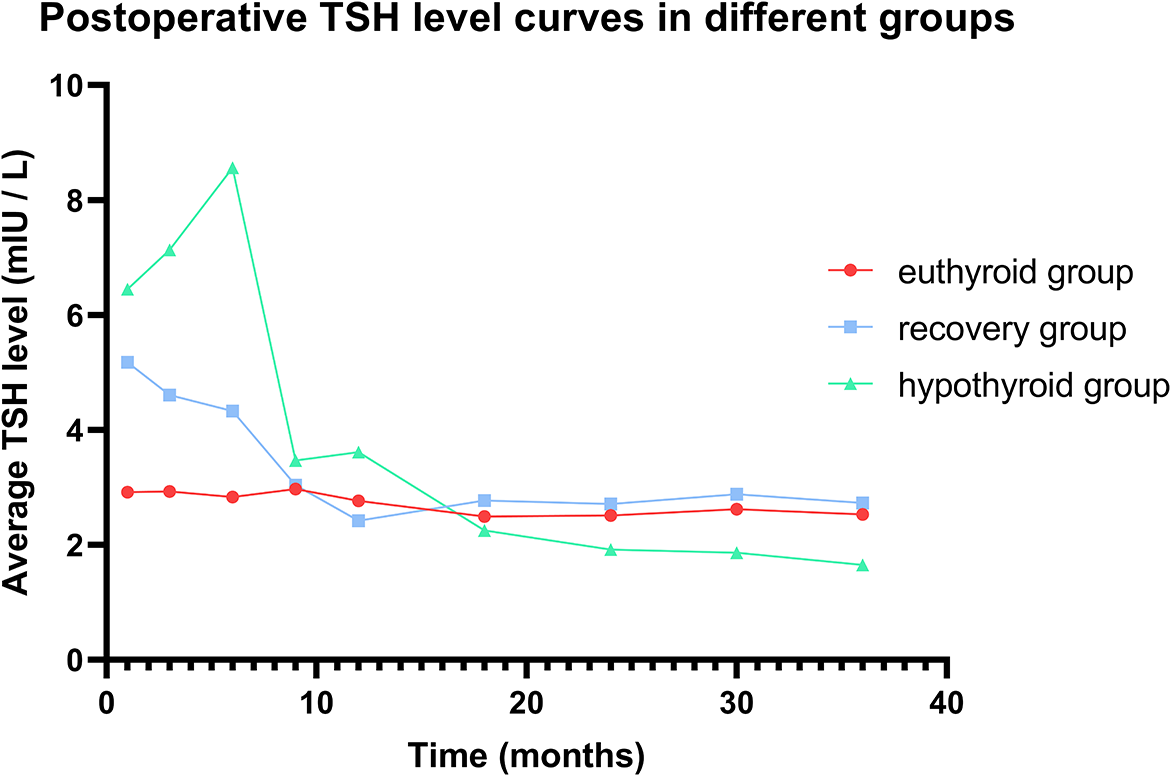
Postoperative TSH level curves in different groups.

**Figure 4:**
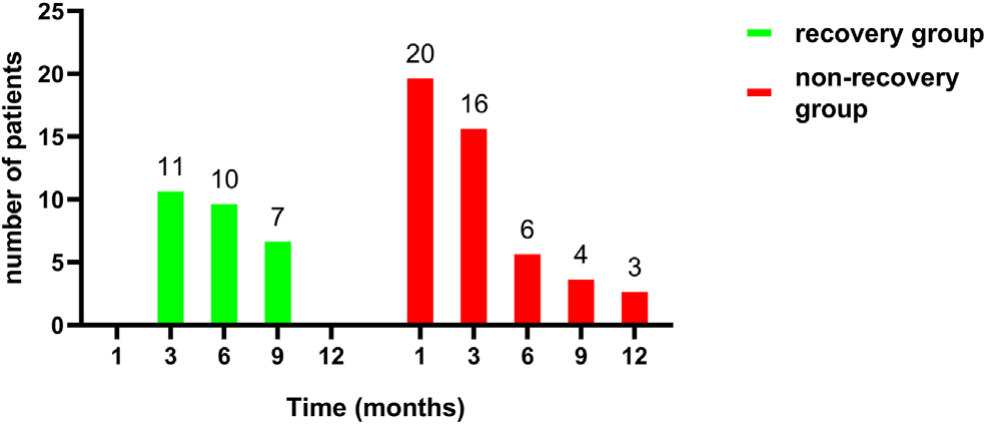
Comparison of distributions between recovery (n=28) and non-recovery group (n=49) per visit month.

We evaluated the postoperative changes in TSH in the different groups during the follow-up period (**Figure 3**). During the follow-up period, both the euthyroid group and recovery group maintained a relatively stable TSH level similar to their preoperative TSH level. The hypothyroid group showed a sharp fluctuation in TSH level due to the supplement therapy. There were no significant correlations between the recovery group and the euthyroid group according to Pearson correlation analysis (P=0.33). **Figure 4** shows the proportion of the two groups with thyroid function recovery and supplement therapy at each visit. The thyroid function of 11 (39.29%) patients had recovered in the third month, followed by 10 (35.71%) in the 6th month and 7(25.00%)in the 9th month. Moreover, no patient recovered in the last month. No patients from the recovery group developed hypothyroidism again.

## Discussion

Many studies have attempted to determine the proportion of patients with hypothyroidism after thyroid lobectomy. In a 35.7-month follow-up study reported by Jin Seong Cho et al. (16), 21.1% of patients developed hypothyroidism after lobectomy. Suyeon Park et al. (11) reported that 35% of patients with hypothyroidism recovered. Amanda Johner et al. (17) reported that 69.2% of patients recovered. In our study, among patients who had hypothyroidism after lobectomy, 38 (28/77, 36.36%) patients spontaneously recovered to the euthyroid state. Above all, 141 (141/190, 74.21%) patients were in the euthyroid state at 24–36 months after follow-up, and these patients were more likely than the other patients to avoid hormone replacement. Further follow-up observational studies are ongoing.

TSH is the main stimulator of thyroid tissue growth and secretor of thyroxine. No guidelines (3, 4) providing a clear recommendation for the follow-up time of postoperative thyroid function are available. Many institutions provide hormone therapy to patients immediately after surgery, which may not be helpful for the recovery of thyroid function (18). In this study, the third month was the peak time of thyroid function recovery. We believe that the recovery of thyroid function might occur mainly in the first year after lobectomy because no patients recovered in the 12^th^ month of follow-up. This is in line with previous studies (19, 20). Lower preoperative TSH levels were associated with thyroid function recovery by univariate analysis. The ability to recovery might be related to lower TSH levels and the degree of lymphocytic infiltration (21) according to previous studies.

TSH suppression therapy for PTC with a low risk of recurrence is controversial (22). A score-matched cohort study reported by Suyeon Park et al. (23) showed no significant difference in DFS between the thyrotropin suppression group and no suppressive group (P=0.57). The ATA guidelines (4) suggest that low-risk patients with TSH < 2 mIU/L should avoid TSH suppression therapy. In addition, considering the excellent prognosis of PTC, the side effects of long-term iatrogenic hyperthyroidism should be considered. In 2013, a prospective study (24) described the association between TSH levels and the incidence rate of cardiovascular diseases. With the inclusion of 524 patients in the experimental group and 1,572 patients in the control group, for each 10-fold reduction in TSH level, the risk of cardiovascular disease mortality increased 3.1-fold. Bone loss and fracture risk were also associated with TSH suppressive therapy, especially in women (25). Therefore, the potential clinical benefits and side effects of TSH suppression need to be balanced. In our follow-up period, no recurrence occurred in those patients, indicating that TSH suppression is not required for low-risk PTC patients. We selected patients with negative pathologic lymph nodes (pN0) shown through central lymph node intraoperative frozen biopsy to reduce the potential risk of recurrence and metastasis with preservation of the contralateral thyroid lobe. Since TSH suppression therapy is not routinely given, unilateral lobectomy could maximize the benefit to patients.

The association between postoperative hypothyroidism and preoperative TSH has been reported by many researchers (9, 19, 26). Jin Seong Cho et al. (16) found that the cut-off value for the preoperative TSH level in predicting postoperative hypothyroidism was 2.0 mIU/L. High preoperative TSH levels had an OR of 2.82 in predicting postoperative hypothyroidism in a cohort study (11). In our study, a high preoperative TSH level was the most independent factor of postoperative hypothyroidism (OR = 12.567, 95% CI = 6.009–26.285; P<0.001). In addition, a low preoperative TSH level was significantly associated with the recovery of thyroid function. Preoperative TSH levels can reflect patients’ thyroid function and thyroid hormone storage. Patients with a high TSH level may have potential thyroid functional defects, thus preventing compensation by the residual thyroid after lobectomy.

Histological HT, the most common autoimmune disease (27), was the second predictor of postoperative hypothyroidism. Peng Ng et al. (28) reported that HT was the only independent risk factor in a retrospective review of 901 patients. The presence of thyroid antibodies was found to be a significant factor by univariate analysis but did not maintain independence as a factor for postoperative hypothyroidism by multivariate analysis. Thyroid antibodies might be associated with the disease mechanism and progression of HT. We speculate that when HT is tested at only the serological level, which means the disease may be mild and that thyroid function can still be compensated after the operation, hypothyroidism may occur later. When thyroiditis is detected by histology, which indicates later disease progression and the likely involvement of the contralateral residual thyroid, hypothyroidism may occur in the early postoperative period. For both serologic and pathological thyroiditis, hypothyroidism may inevitably occur after operation, and the benefit of lobectomy will be decreased. Furthermore, although still controversial, some articles suggest that HT is associated with the risk of developing thyroid cancer (29, 30). Therefore, we suggest total thyroidectomy for patients with HT.

Interestingly, we found that right-side lobectomy was an independent factor associated with postoperative hypothyroidism (OR 2.404; 95% CI 1.139–5.077; P=0.021), which is consistent with the present research (11). Some studies (31, 32) have considered the difference in size of the left and right lobes of the thyroid gland. The right thyroid lobe is usually larger in volume than the left. This asymmetry in thyroid size might result from differences in handedness. This asymmetry led to a significant risk for hypothyroidism when the residual thyroid tissue was small, as demonstrated by other reports (33). However, there may be some differences in blood supply and endocrine function between the left and right lobes of the thyroid gland. A large-sample study of 299,908 patients from Japan (34) showed that right lobe thyroid hemiagenesis was more common than left lobe thyroid hemiagenesis, a finding that requires further research regarding the embryology and physiology of the thyroid lobes.

There were several limitations in this study. First, we did not strictly restrict the diet of the study cohort (such as iodine intake), which may have had an impact on the laboratory test results. Second, considering the excellent prognosis of PTC, further research is underway with a longer follow-up time to verify the recovery time of thyroid function, recurrence rate, DFS, and overall survival. Third, unfortunately, our data did not include preoperative or postoperative residual thyroid volume. However, this was a unique study of low-risk PTC patients after unilateral lobectomy performed to identify the proportion of patients who benefited from lobectomy when interference from hormone supplementation was excluded.

In conclusion, our study indicated that 40.53% of PTC patients developed temporary or permanent hypothyroidism. Overall, 74.21% of patients were in the euthyroid state and likely to avoid hormone replacement. Postoperative hypothyroidism was independently associated with a higher level of preoperative TSH, HT, and right lobe lobectomy. TSH level were found to be related to the recovery of hypothyroidism. We suggest that low-risk patients be followed for at least one year to develop an individual thyroid function management strategy. Patients with lower preoperative TSH levels, without Hashimoto’s thyroiditis, and with left lobe lobectomy might benefit more from lobectomy.

## Data Availability Statement

The raw data supporting the conclusions of this article will be made available by the authors, without undue reservation.

## Ethics Statement

The studies involving human participants were reviewed and approved by Medical Ethics Committee of The First Affiliated Hospital of Chongqing Medical University. Written informed consent for participation was not required for this study in accordance with the national legislation and the institutional requirements.

## Author Contributions

The first author of this manuscript is YD. YD, DH, and XS designed this research. YC and DH collected the data and performed the statistical analyses. YD and XS reviewed the results, interpreted the data, and wrote the manuscript. YD, YC, and DH discussed and edited the paper. All authors contributed to the article and approved the submitted version.

## Conflict of Interest

The authors declare that the research was conducted in the absence of any commercial or financial relationships that could be construed as a potential conflict of interest.
